# A Robust Superhydrophobic Polyurethane Sponge Loaded with Multi-Walled Carbon Nanotubes for Efficient and Selective Oil-Water Separation

**DOI:** 10.3390/nano11123344

**Published:** 2021-12-09

**Authors:** De Liu, Shiying Wang, Tao Wu, Yujiang Li

**Affiliations:** 1 Shandong Provincial Research Center for Water Pollution Control, School of Environmental Science and Engineering, Shandong University, Qingdao 266237, China; 201912689@mail.sdu.edu.cn; 2 Key Laboratory of Colloid and Interface Science of Education Ministry, Shandong University, Jinan 250100, China; 201912688@mail.sdu.edu.cn

**Keywords:** superhydrophobic, PU sponge, wettability, oil-water separation, multi-walled carbon nanotubes

## Abstract

The influence of different coupling agents and coupling times on the wettability of a polyurethane (PU) sponge surface were optimized. Octadecyltrichlorosilane (OTS) was selected as the optimal coupling agent to prepare the superhydrophobic sponge. The superhydrophobic sponge was prepared in one step, which has the advantages of simple operation and enhanced durability. The superhydrophobic sponge was characterized by scanning electron microscopy, Teclis Tracker tensiometry, and Fourier transform infrared (FT-IR) spectrophotometry. The water contact angle increased from 64.1° to 151.3°, exhibiting ideal superhydrophobicity. Oils and organic solvents with different viscosities and densities can be rapidly and selectively absorbed by superhydrophobic sponges, with an absorption capacity of 14.99 to 86.53 times the weight of the sponge itself, without absorbing any water. Since temperature affects the viscosity and ionic strength of oil, and influences the surface wettability of the sponges, the effect of temperature and ionic strength on the oil absorption capacity of the superhydrophobic sponges was measured, and its mechanism was elucidated. The results showed that the absorptive capacity retained more than 90% of the initial absorptive capacity after repeated use for 10 times. Low-cost, durable superhydrophobic sponges show great potential for large-scale oil-water separation.

## 1. Introduction

With the economic growth, the development of the petroleum industry and marine transportation have made marine oil spills a frequent and severe environmental challenge [1,2]. The spilled oil will eventually be consumed by humans after accumulating in farmed fish and shellfish, posing a major risk to human health, as well as to the aquatic environment [3,4]. Therefore, effective oil-water separation is urgently necessary. Various methods have been used for remediating oil spills, such as surface skimming [5,6,7], in situ burning [8] dispersants [9], and absorption. Due to its simple operation and easy separation, the absorption method is usually used for oil-water separations. Traditional absorbent substances such as cellulose [10,11], activated carbon [12,13] and zeolites [14], are mostly microporous materials. These materials therefore have a very low adsorption capacity, resulting in unsatisfactory oil-water separation efficiency. Therefore, researchers have attempted to find inexpensive and effective oil-water separation materials. 3D adsorbent materials possess the characteristics of large pores and large specific surface area, which can absorb oil on the material surface and realize oil-water separations [15]. In addition, they are not only inexpensive, but also have a large absorption capacity [16]. However, since 3D adsorbent materials’ surface wettability is typically hydrophilic, they have an unsatisfactory hydrophobic oil absorption performance. Specifically, their oil absorption efficiency and oil-water separation efficiency are usually sub-optimal [17]. As a consequence, researchers have been searching for materials with a hydrophobic surface or a superhydrophobic surface, and a good affinity for hydrophobic oil droplets, for efficient oil-water separation.

A material with a water contact angle greater than 150° is termed a superhydrophobic material [18,19,20]. Superhydrophobic materials can be obtained by simultaneously increasing the ruggedness of the material surface and reducing the surface free energy [21]. Three-dimensional porous materials with superhydrophobic/superlipophilic properties have received widespread attention because they can selectively absorb oil [22]. For example, polyurethane sponge is a 3D porous material that possesses excellent characteristics, such as low density, low price, superior elasticity, and high absorption capacity [23]. However, since sponges have hydroxyl and carboxyl groups on their surfaces, which are usually hydrophilic, surface modification is required [24]. For example, Qiu et al. [25] used one-step ultrasonic dip-coating method to obtain a superhydrophobic sponge. This sponge can selectively absorb a variety of oils and nonpolar solvents. Wu et al. [16] fabricated a superhydrophobic POS@HNT-PUF by the grafting of Hexadecyltrimethoxysilane (HDTMS) and Tetraethyl orthosilicate (TEOS), which can be used for oil-spill cleanup. In addition, some other nanomaterials, such as SiO_2_ nanoparticles [26], Fe_3_O_4_ nanoparticles [27,28], nanodiamonds [29] and reduced graphene oxide [23,30,31] have also been used to modify sponges. Multi-walled carbon nanotubes (MWCNTs) are used in various applications due to their outstanding electrical, optical, and mechanical properties [32,33]. In addition, MWCNTs can be used as an adsorbent material due to their high aspect ratio, low density, and good environmental chemical stability [34]. Hydroxylated multi-walled carbon nanotubes (MWCNTs–OH) are chemically functionalized materials with hydroxyl groups on their surface, making them easy to chemically modify [35,36]. However, since pure MWCNTs are one-dimensional solid materials with no oil storage space, they cannot be used directly for oil absorption.

In this paper, three kinds of hydrophobic long-chain siloxanes were selected to modify both carbon nanotubes and sponges, and the hydrophobic sponges were obtained by a one-step method. The hydrophobic sponge was characterized by Fourier transform infrared (FT-IR) spectrophotometry, scanning electron microscope, and interface rheometer. The effects of temperature and ionic strength on oil absorption efficiency of superhydrophobic sponges were also investigated. Several oils and organic solvents were adopted as model pollutants, and the absorption mechanism was identified and discussed.

## 2. Experimental Section

### 2.1. Materials

About 50 μm long hydroxyl multi-walled carbon nanotubes (95%), with an outer diameter of 8–15 nm and inner diameter of 3–5 nm were procured from Xfnano Materials Tech Co., Ltd. (Nanjing, China). Octadecyltrichlorosilane (OTS) with purity of 98% was procured from the Shanghai McLean Chemical Reagent Co., Ltd. (Shanghai, China). Polyurethane (PU) sponges were acquired from Top Daily Chemicals Co., Ltd. (Wuxi, China). Octadecyldimethyltrimethoxysilylpropylammonium chloride solution (C_26_H_58_ClNO_3_Si, 60 wt. % in methanol) and hexadecyltrimethoxysilane (H_3_C(CH_2_)_15_Si (OCH_3_)_3_, ≥85%) were from Aladdin Reagents Co., Ltd. (Shanghai, China). Sulfuric acid (H_2_SO_4_, 98.0%) was acquired from Laiyang Kan gde Chemical Co., Ltd. (Laiyang, China). Hydrogen peroxide (70%) was acquired from Sinopharm Chemical Reagent Co., Ltd. (Shanghai, China). Various chemical reagents, including acetone, toluene, anhydrous ethanol, CCl_4_, and NaCl, etc., were all of analytical grade.

### 2.2. Pretreatment of Original PU Sponge

After ultrasonic cleaning of PU sponge with acetone, absolute ethanol, and distilled water for 20 min, respectively, it was put in a drying oven at 80 °C for 24 h. Then the cleaned sponge was oxidized with H_2_O_2_/H_2_SO_4_ solution to make its surface rich in hydroxyl groups, which was easy to modify later. 70% H_2_O_2_ was mixed with H_2_SO_4_ solution of 49% concentration in a volume ratio of 7:3. The washed PU sponge was soaked in the H_2_O_2_/H_2_SO_4_ solution for 4 h, and then it was cleaned with distilled water. The pre-treated sponges were put in a vacuum drying oven at 80 °C for 24 h.

### 2.3. Preparation of Coupling Agent-CNTs/PU Sponges

Fifty mg of hydroxylated MWCNTs were slowly added to 50 mL of toluene, and the carbon nanotubes were dispersed by ultrasonication for 20 min. Subsequently, the coupling agent was slowly added to prepare a liquid with a concentration of 0.05 mol/L. The pre-treated PU sponge was immersed in the modified CNTs/toluene solution, and the compound was magnetically agitated at 300 rpm for 24 h. The mixed system was ultrasonicated for 20 min, and then allowed to stand for 2 h. Eventually, the coupling agent-CNTs/PU sponge was removed, then was placed in a drying oven and dried at 80 °C for 24 h.

### 2.4. Characterization

The static water contact angle (WCA) was observed on a Teclis Tracker tensiometer (Teclis Instruments, Civrieux d’Azergues, France). Scanning electron microscopy (SEM) images of sponges were acquired on a JSM-6330F scanning electron microscope (JEOL Ltd., Tokyo, Japan). The ζ-potential of the sponges was assessed by a SurPASS analyzer (Anton Paar, Graz, Austria). Fourier transform infrared (FT-IR) spectrophotometry of the pristine and modified PU sponges were obtained on an AXS FT-IR spectrophotometer (Bruker, Karlsruhe, Germany), with KBr used as the dispersion medium. The viscosity of the oil at different temperatures was measured by a HAAKE RS75 Rheometer (Thermo Fisher Scientific, Waltham, MA, USA). Thermogravimetric analysis (TGA) curves were recorded using a TGA5500 system (TA Instruments Co. Ltd., New Castle, DE, USA) under a nitrogen atmosphere by heating from 20 to 500 °C at a heating rate of 10 °C/min.

### 2.5. Oil Absorption of the OTS-CNTs/PU Sponge to Different Oils

The absorption capacity of OTS-CNTs/PU sponge on soybean oil, kerosene, chloroform, petroleum ether, hexadecane, and crude oil was tested. The oil absorption test conditions of the sponge were as follows: the sponge was soaked in oil for 5 min, and then taken out and weighed. The following formula was used to calculate the absorption capacity:

(1) $$k=\frac{m_{p}-m_{o}}{m_{o}}$$

where *m_p_* and *m_s_* are the mass of OTS-CNTs/PU sponges prior to and after adsorption, separately. The results of each group were measured three times and averaged.

## 3. Result and Discussion

### 3.1. Preparation of Superhydrophobic Sponge

#### 3.1.1. Selection of Coupling Agents

**Figure 1:**
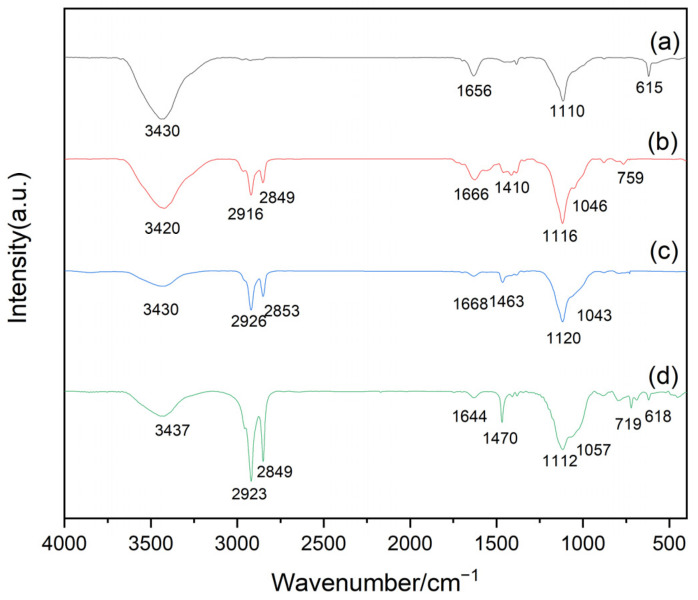
Fourier transform infrared (FT-IR) spectrophotometry of: (a) pre-treated polyurethane (PU) sponge, (b) octadecyldimethyltrimethoxy-carbon nanotubes (CNTs)/PU sponge, (c) hexadecyltrimethoxylsilane-CNTs/PU sponge, and (d) octadecyltrichlorosilane (OTS)-CNTs/PU sponge.

Octadecyldimethyltrimethoxysilylpropylammonium chloride, hexadecyltrimethoxysilane and octadecyltrichlorosilane (OTS) were selected as coupling agents, which fully reacted with the hydroxyl groups on the carbon nanotubes and PU sponges under the condition of magnetic stirring for 24 h. The coupling agent was grafted on the surfaces of the hydroxylated multi-walled carbon nanotubes and PU sponges. Then, the carbon nanotubes were loaded on the PU sponge by the ultrasonic method. The FTIR spectra of various coupling agent-CNTs/PU sponges are displayed in Figure 1. The peak at 3430 cm^−1^ in the pre-treated PU sponge spectrum was ascribed to the stretching vibration peak of the –OH group [22]. The bands at 1110 cm^−1^ were assigned to the C–O stretching vibration [18]. The adsorption band at 1656 cm^−1^ corresponded to the presence of the C=C bonds [37]. The adsorption bands at 615 cm^−1^ were ascribed to –SO₃H. After modification, the FT-IR spectrum of the coupling agent-CNTs/PU sponge exhibited certain changes. For instance, the FT-IR spectrum of the OTS-CNTs/PU sponges showed four additional absorption peaks at 2923, 2849, 1470 and 719 cm^−1^, corresponding to the stretching vibration and bending vibration of the long-chain alkyl methylene C−H [38]. Additionally, the adsorption band at 1112 and 1057 cm^−1^ were ascribed to Si−O−Si and Si−O−C, respectively [37,39]. The FT-IR spectra of other coupling agent-CNTs/PU sponges had similar peaks, indicating that the hydroxyl groups reacted with the coupling agents.

**Figure 2:**
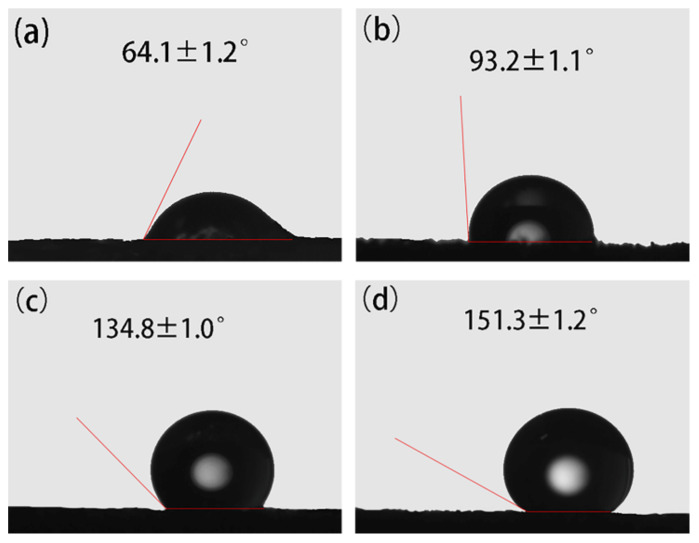
Water contact angles (WCAs) of various coupling agent-CNTs/PU sponges: (**a**) pre-treated PU sponge, (**b**) octadecyldimethyltrimethoxysilylpropylammonium chloride-CNTs/PU sponge, (**c**) hexadecyltrimethoxysilane-CNTs/PU sponge, and (**d**) Octadecyltrichlorosilane (OTS)-CNTs/PU sponge (experimental conditions: pH = 7, coupling time = 24 h at 25 °C).

Wettability is a solid-liquid interface property [21]. The wettability was judged by performing water contact angle tests on three modified sponges [40]. It can be seen from Figure 2a that the WCAs of the pre-treated PU sponges are 64.1 ± 1.2°, which is hydrophilic. The hydrophilicity of the pre-treated sponge is ascribed to the hydroxyl on the surface. The WCAs of the prepared sponges were also measured, and the modified sponges give WCAs of 93.2 ± 1.1°, 134.9 ± 1.0°, and 151.3 ± 1.2° for octadecyldimethyltrimethoxysilylpropylammonium chloride-CNTs/PU sponge, hexadecyltrimethoxysilane-CNTs/PU sponge, and octadecyltrichlorosilane (OTS)-CNTs/PU sponge (OTS-CNTs/PU sponge), respectively, which means that the modified sponges are harder to be wet with water (Figure 2b–d). Among them, OTS-CNTs/PU has the largest WCAs, which is higher than 150°, completely repels the water, and thus exhibits markedly ideal superhydrophobicity.

**Figure 3:**
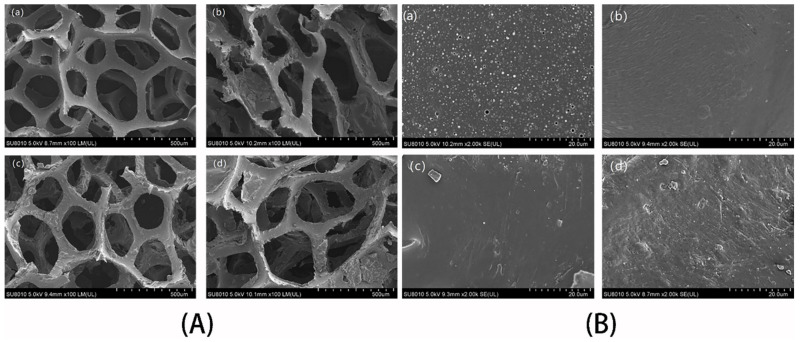
Low magnification (**A**) and high magnification (**B**) Scanning electron microscopy (SEM) images of (**a**) pre-treated sponge, (**b**) octadecyldimethyltrimethoxysilylpropylammonium chloride-CNTs/PU, (**c**) hexadecyltrimethoxylsilane-CNTs/PU, and (**d**) OTS-CNTs/PU with coupling time = 24 h.

To study the superficial morphology of the pre-treated PU sponges and the modified PU sponges, scanning electron microscope (SEM) tests were performed. Low magnification images of the pre-treated PU sponges and modified sponges are presented in Figure 3A. The pristine PU sponges have a unique 3D layered porous structure, with a pore size between 200–500 microns, and the large pore structure of the sponge gives it more space to store oil. Extant literature has demonstrated that nanomaterials with low surface free energy are easy to uniformly adhere to the sponge skeleton without destroying its inherent structure [26,41]. As shown in Figure 3A(c,d), the OTS-CNTs/PU sponge and hexadecyltrimethoxysilane-CNTs/PU sponge maintained the original 3D porous structure, indicating that its absorption capacity did not decrease. However, the skeleton structure of the octadecyldimethyltrimethoxysilylpropylammonium chloride-CNTs/PU sponge was damaged to a certain extent.

Figure 3B provides higher magnification images. As shown in Figure 3B, there is a small number of carbon nanotubes on the octadecyldimethyltrimethoxysilylpropylammonium chloride-CNTs/PU sponges, while there are more carbon nanotubes on the hexadecyltrimethoxylsilane-CNTs/PU sponges and OTS-CNTs/PU sponges. Among them, the OTS-CNTs/PU sponge has a rougher surface and stronger hydrophobicity. In addition, longer alkyl chains lead to greater hydrophobicity [42]. Therefore, octadecyltrichlorosilane was selected as the coupling agent.

#### 3.1.2. Selection of Coupling Time

**Figure 4:**
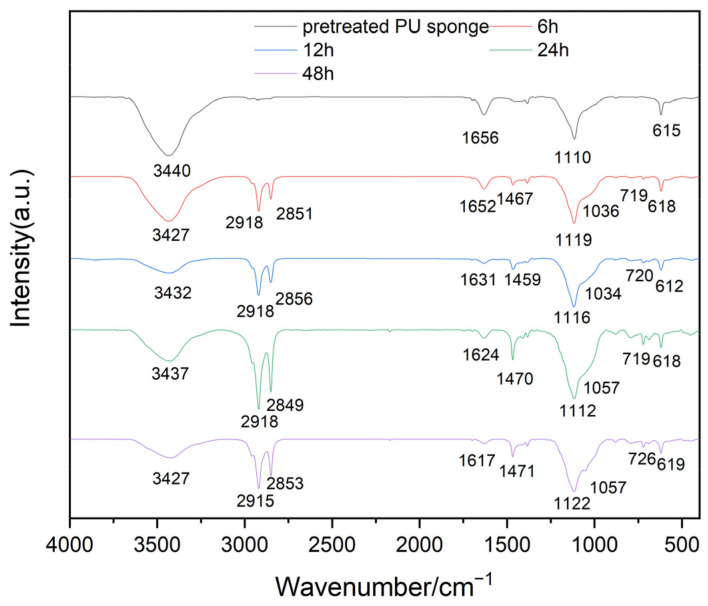
FT-IR spectra of OTS-CNTs/PU sponge with different coupling times.

The sponges loaded with carbon nanotubes with a coupling time of 6 h, 12 h, 24 h, and 48 h were selected for FT-IR analysis. As indicated in Figure 4, the intensity of the peaks of Si−O−Si and CH_2_ increased with coupling time.

**Figure 5:**
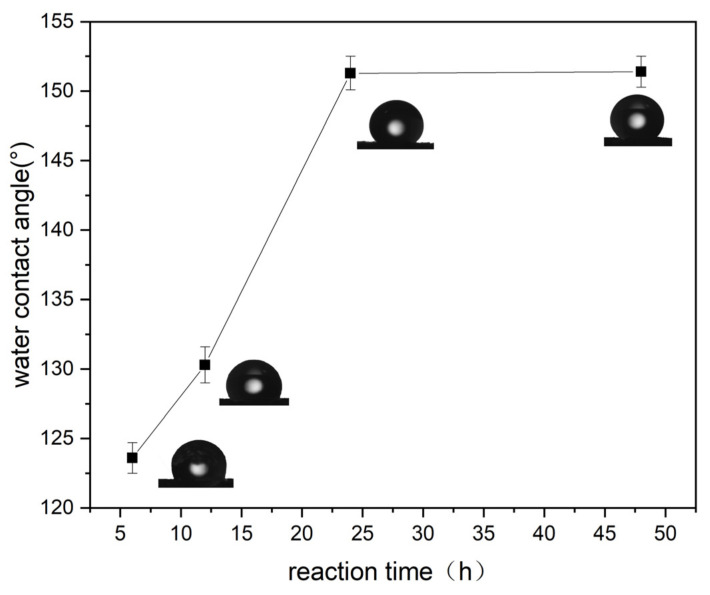
Effect of coupling time on the WCA (experimental conditions: pH = 7, coupling time = 24 h at 25 °C).

As indicated in Figure 5, wettability was measured by water contact angle test. As the coupling time increased, the water contact angle increased, indicating that its hydrophobicity increased. This is because the longer is the coupling time, the fuller is the reaction between carbon nanotubes and OTS. When the coupling time was 24 h, the water contact angle was 151.3 ± 1.2°, which is superhydrophobic. When the coupling time changed from 24 h to 48 h, the water contact angle did not change significantly, indicating that the carbon nanotubes and OTS had reacted completely.

**Figure 6:**
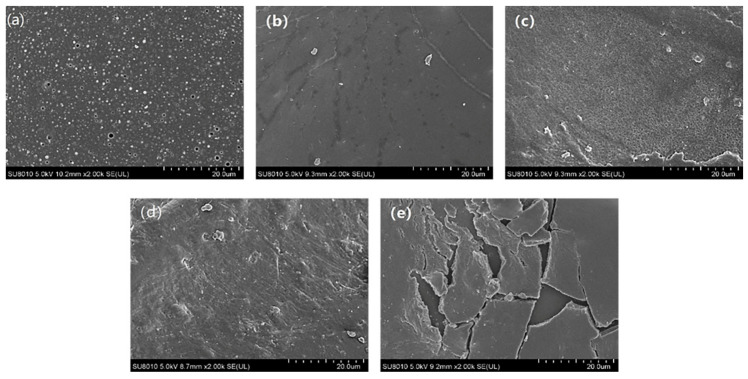
High magnification SEM images of OTS-CNTs/PU with different coupling times (**a**) pre-treated sponge, (**b**) 6 h, (**c**) 12 h, (**d**) 24 h, and (**e**) 48 h.

Figure 6 presents SEM diagrams of different coupling times. As the coupling time increased, the number of carbon nanotubes loaded on the sponge surfaces increased, thus increasing the surface ruggedness. The increase in surface roughness facilitated the enhancement of hydrophobicity. Therefore, considering both the wettability and the adsorption capacity of the sponge, the OTS-CNTs/PU sponge with a coupling time of 24 h was selected.

### 3.2. Oil Absorption Test

#### 3.2.1. Oil-Water Separation Process

**Figure 7:**
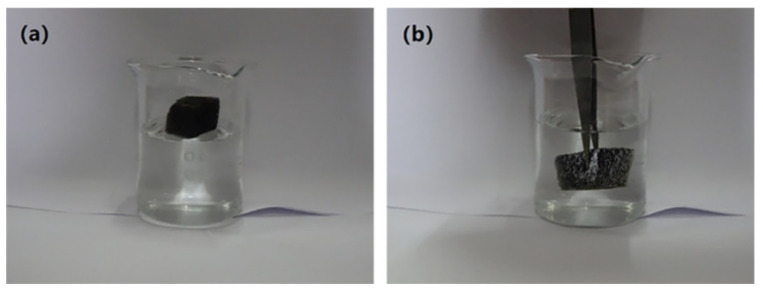
(**a**) Optical image of OTS-CNTs/PU sponge floating on the water surface; (**b**) optical image of OTS-CNTs/PU sponge immersed in a water bath under external force.

The OTS-CNTs/PU sponge could float when placed on water surface (Figure 7a). As indicated in Figure 7b, as the superhydrophobic sponge was dipped in water with forceps, a silvery, mirror-like film of air formed around its surface, which shows that the sponge possessed the surface characteristics of Cassie Baxter [43]. Because of this air film, the superhydrophobic sponge cannot be infiltrated by water. When the tweezers were removed, the superhydrophobic sponge promptly floated onto the water surface and did not increase in weight, indicating that it had not absorbed water.

**Figure 8:**
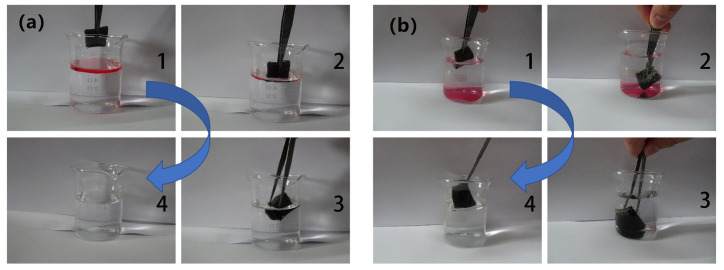
(**a**) Optical images of the removal process of hexadecane from the water surface using OTS-CNTs/PU sponge; (**b**) optical images of the removal process of chloroform from underwater using OTS-CNTs/PU sponge (The blue arrow shows the sequence 1–4 of oil absorption process).

In this study, hexadecane and chloroform were adopted for the absorption process observation experiment. As indicated in Figure 8a, oil-red stained hexadecane was added on water, and then the OTS-CNTs/PU sponge was dipped on the immiscible oil-water mixture. In seconds, the oil was completely absorbed by the OTS-CNTs/PU sponge. When the sponge was clamped away with a tweezer, a clear and transparent water surface was obtained. Figure 8b shows the sponge absorbing chloroform (dyed with oil red) from underwater. Immersion of OTS-CNTs/PU sponge into water can form a three-phase sponge-air-water interface. The super-hydrophobicity of the sponge kept water out of the sponge. When chloroform was in proximity to the superhydrophobic sponge, it was promptly absorbed into the sponge. The oil-water selective separation process was finally completed in a short period of time.

#### 3.2.2. Impact of Environmental Factors on the Efficiency of Oil-Water Separation

Since oil and organic solvent spills usually occur under complex environmental conditions, it is crucial to elucidate the performance of superhydrophobic sponges under various conditions [44,45]. The oil absorption efficiency of superhydrophobic sponges can be affected by changes in environmental factors. For example, temperature can influence the viscosity of the oil, and ionic strength can affect the apparent wettability of the superhydrophobic sponges, thereby affecting the oil absorption efficiencies of the superhydrophobic sponges.

##### Effect of Density and Viscosity

When oil is absorbed in the pores of the superhydrophobic sponge by capillary force, it fills the volume of the pores. Under the action of the hydrophobic force, the hydrophobic oil will also be adsorbed on the outer surface of the sponges. As seen in Table 1, the absorption capacities of the six different oils vary from 14.99 times to 86.53 times, which is attributable to the different viscosity, density, and other properties of the oils [46]. The amount of oil that can be stored per unit volume of the sponge composite increases as the density of the oil increases. At the same time, for high viscosity oil, the high viscosity prevents it from diffusing into the internal pores of the adsorbent, resulting in ineffective oil capture [47]. The oil absorption capacity of the sponge increases after modification (Table 2), which is related to its enhanced hydrophobicity.

##### Effect of Temperature

Increasing the oil temperature can reduce the oil viscosity [48,49], which can make the oil more fluid [50]. Temperature can alter the oil viscosity and fluidity, thereby affecting the oil absorption efficiency of the superhydrophobic sponges.

Chloroform, crude oil, and kerosene were used to determine the influence of temperature on the oil absorption of superhydrophobic sponges. As presented in Table 3, the oil absorption of sponges at 20, 40, 60 and 80 °C were measured, and the oil viscosity at different temperatures when the shear rate was 1 s^−1^ was measured. As the temperature increased, the viscosity of various oils decreased. For high viscosity oils, the reduced viscosity causes the oil to be more easily absorbed by the sponge. But for the lower viscosity oil, the lower viscosity causes its adhesion on the sponge skeleton to decrease.

##### Effect of Ionic Strength

Surface wettability of superhydrophobic sponges can be affected by ionic strength, which in turn influences the oil absorption capacity. The ionic strength can be changed by adding electrolytes (e.g., NaCl and CaCl_2_). The OTS-CNTs/PU sponges were soaked in different concentrations of NaCl or CaCl_2_ solution for 24 h, and their WCAs were measured after drying. Subsequently, their absorption of different oils was assessed. It can be seen from Table 4 that as the ionic strength increased, the WCA of the OTS-CNTs/PU sponge decreased, indicating that the hydrophobicity decreased. Moreover, the oil absorption of the sponge composite material will also decrease. This is because the wettability of the sponge changed from superhydrophobic to hydrophobic, resulting in a reduction in the hydrophobic force between the sponge and the oil droplets. The effects of the two salts on the sponge composites were also compared, and it was found that the same concentration of CaCl_2_ had a greater impact on the hydrophobicity and oil absorption of OTS-CNTs/PU sponges than did NaCl. The zeta potential of the superhydrophobic sponge is −20.78 mV. Ca^2+^ and Na^+^ can be adsorbed to the surface of the sponge through electrostatic action, and they are easily combined with water molecules in the air and liquid phase through hydrogen bonds, resulting in the reduction of water contact angle and the change of surface wettability from superhydrophobic to hydrophobic, which in turn affects the absorption efficiency. The interaction between Ca^2+^ and water molecules is greater than the interaction between Na^+^ and water molecules.

### 3.3. Reusability of the Superhydrophobic Sponge

**Figure 9:**
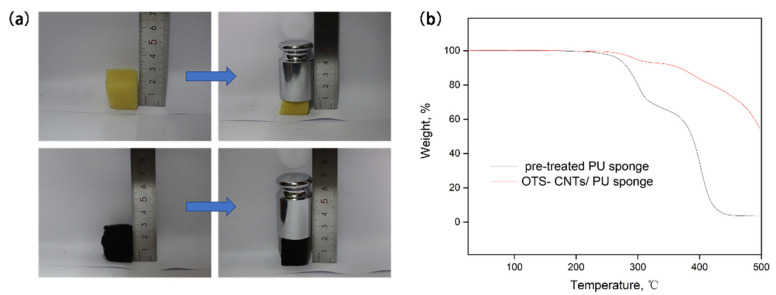
(**a**) Shape of PU sponge and OTS-CNTs/PU sponge under the same external force; (**b**) Thermogravimetric analysis (TGA) curves of pre-treated PU sponge and OTS-CNTs/PU sponge.

Due to the grafting of OTS with MWCNTs, the weight of the modified sponge was increased by about 33%. CNTs possess unique mechanical toughness [51]. Therefore, when a 200 g weight was placed on a 20 mm × 20 mm × 20 mm pristine PU sponge and superhydrophobic sponge, the PU sponge was severely squashed and deformed, whereas, the OTS-CNTs/PU sponge could remain undeformed due to its excellent mechanical strength (Figure 9). This shows that the OTS-CNTs/PU sponge has unique mechanical strength. The enhancement of mechanical strength is ascribed to the mechanical enhancement of carbon nanotubes loaded on the skeleton of the sponges [18]. The thermal stability of pre-treated sponges and OTS-CNTs/PU sponges was investigated by TGA curves [52,53]. As shown in Figure 9b, the pre-treated sponge decomposed almost completely at 350–450 °C, and the residual weight of 3.5% at 500 °C. In contrast, the residual weight of OTS-CNTs/PU sponge at 500 °C was 54.65%, indicating its improved heat resistance after modification.

**Figure 10:**
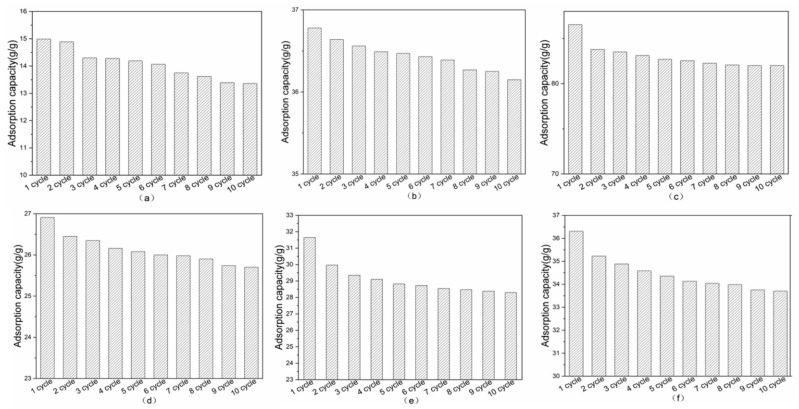
Variations in absorption capacities of OTS-CNTs/PU sponge for various oils under 10 cycles: (**a**) soybean oil; (**b**) kerosene; (**c**) chloroform; (**d**) petroleum ether; (**e**) hexadecane; and (**f**) crude oil.

The adsorption performance of OTS-CNTs/PU sponges was examined by performing 10 replicate adsorption/desorptions of six kinds of oils. As seen in Figure 10, the oil absorption ability of OTS-CNTs/PU sponges decreased slightly during 10 cycles. This is because the recycling of the sponge composite material is realized through the mechanical extrusion process. However, the oil absorbed by the material cannot be completely squeezed out during the mechanical extrusion process, and the oil will remain in the pores of the sponge, thus increasing its weight [54]. Furthermore, the extrusion process will cause irreversible deformation of OTS-CNTs/PU sponges, which will reduce the oil storage space of the sponge. Several reported oil-absorbing materials were compared (Table 5) and OTS-CNTs/PU sponges showed good performance.

## 4. Conclusions

In this study, different coupling agents and coupling times were selected to prepare superhydrophobic sponges. Finally, octadecyltrichlorosilane was used as the optimal coupling agent to prepare the optimum superhydrophobic sponge. The introduction of long-chain alkyl groups makes the sponge superhydrophobic and increases the water contact angle from 64.1° to 151.3°. The superhydrophobic sponge is prepared by a one-step method, so that OTS can react not only with hydroxyl groups on CNTs, but also with hydroxyl groups on pretreated sponges, making the prepared sponges more durable. Six oils with different viscosities and densities were selected as model pollutants for the absorption capacity test. The superhydrophobic sponge possesses excellent mechanical properties and large absorption capacity, which can absorb oil that is 14.99 to 86.53 times its own weight. In addition, it can be reused 10 times by squeezing, and its absorption capacity does not decrease significantly. The influence of temperature and ionic strength on it was also measured, and it was found that the absorption of oil by the superhydrophobic sponge is based on capillary force and hydrophobic force. Therefore, the OTS-CNTs/PU sponge constitutes a highly potential candidate for oil spill absorption.

## Figures and Tables

**Table 1:** Absorption capacity of the Octadecyltrichlorosilane (OTS)-carbon nanotubes (CNTs)/polyurethane (PU) sponges for various organic oils and solvents (20 °C).

Samples	Density (g/mL)	Viscosity (mPas)	Absorption Capacity (g/g)
Soybean oil	0.919	76.59	14.99
Kerosene	0.79	3.489	36.78
Petroleum ether	0.705	1.75	26.91
Chloroform	1.484	1.726	86.53
Crude oil	0.835	6.087	36.81
Hexadecane	0.773	7.645	31.65

**Table 2:** Absorption capacity of t different PU sponges for various organic oils and solvents (20 °C).

Absorption Capacity (g/g)	Soybean Oil	Kerosene	Petroleum Ether	Chloroform	Crude Oil	Hexadecane
OTS-CNTs/PU sponge	14.99	36.78	26.91	86.53	36.81	31.65
OTS-PU sponge	14.81	36.60	26.82	86.39	35.66	31.57
Pre-treated PU sponge	14.17	35.66	26.15	85.37	32.68	30.89

**Table 3:** Effect of temperature on oil absorption capacity (shear rate was fixed as 1 s^−1^ to measure viscosity of the oil).

Samples	Temperature (°C)	Viscosity (mPas)	Absorption Capacity (g/g)
Chloroform	20	3.177	86.53
	40	3.063	85.76
	60	2.941	85.62
	80	*	*
Crude oil	20	11.78	36.81
	40	9.276	37.26
	60	7.413	37.91
	80	6.087	38.68
Kerosene	20	3.489	36.79
	40	2.764	35.92
	60	1.697	35.02
	80	1.671	35.00

* Chloroform boils at 80 °C.

**Table 4:** Effect of ionic strength on oil absorption capacity (20 °C).

Samples	c(NaCl)	WCA	Absorption (g/g)	c(CaCl_2_)	WCA	Absorption (g/g)
Chloroform	0	151.3°	86.53	0	151.3°	86.53
	0.002	143.1°	86.39	0.002	142.8°	85.88
	0.004	141.6°	86.12	0.004	140.5°	85.59
	0.006	134.4°	85.76	0.006	132.6°	85.15
	0.008	125.2°	85.12	0.008	120.9°	85.11
Crude oil	0	151.3°	36.81	0	151.3°	36.81
	0.002	143.1°	36.17	0.002	142.8°	35.94
	0.004	141.6°	36.10	0.004	140.5°	35.74
	0.006	134.4°	36.08	0.006	132.6°	35.60
	0.008	125.2°	35.77	0.008	120.9°	35.58
Kerosene	0	151.3°	36.79	0	151.3°	36.79
	0.002	143.1°	36.68	0.002	142.8°	36.63
	0.004	141.6°	36.64	0.004	140.5°	36.4
	0.006	134.4°	36.51	0.006	132.6°	36.07
	0.008	125.2°	36.27	0.008	120.9°	36.06

WCA: water contact angle.

**Table 5:** WCA and oil absorption capacity of different absorbent materials.

Adsorption Material	WCA	Sorbate	Oil Adsorption Capacity (g/g)	Reference
Cellulosic materials	148 °	diesel oil	33	[55]
PU-PNIPAAm	135°	1,2-dibromoethane	11.31	[37]
N-CNS	142°	ethylene glycol	30	[50,56]
CNTs/PUF	131°	chloroform	33.04	[57]
PU–NDs-fPDA sponge	>150°	chloroform	59.26	[29]
CSTN	150°	chloroform	74.32	[58]
RGO/OAP/PU	>150°	chloroform	80.28	[23]
PUf-g-LMA	*	kerosene	20.97	[59]
NCPUF	148°	kerosene	27.7	[60]
OTS-CNTs/PU sponge	150.2°	kerosene	36.78	Present work
OTS-CNTs/PU sponge	150.2°	chloroform	86.53	Present work

* Not mentioned in the reference.

## Data Availability

Data can be available upon request from the authors.
